# Systematic Review and Meta‐Analysis of Patient Experiences of Oesophageal Cancer Survivorship After Oesophagectomy

**DOI:** 10.1002/pon.70482

**Published:** 2026-05-11

**Authors:** Jonathan Sivakumar, Thang Dao, Feras Alnimri, David S. Liu, Qianyu Chen, Rebekah Laidsaar‐Powell, Michael W. Hii, Cuong Phu Duong

**Affiliations:** ^1^ Division of Cancer Surgery Peter MacCallum Cancer Centre Melbourne Australia; ^2^ Department of Surgery The University of Melbourne Melbourne Australia; ^3^ Department of Gastroenterology St Vincent's Hospital Melbourne Fitzroy Australia; ^4^ Department of Medicine The University of Melbourne Melbourne Australia; ^5^ Upper Gastrointestinal Surgery Unit Austin Hospital Heidelberg Australia; ^6^ Department of Surgery Victorian Interventional Research and Trials Unit The University of Melbourne Heidelberg Australia; ^7^ Department of Upper Gastrointestinal Surgery St Vincent's Hospital Melbourne Fitzroy Australia; ^8^ Psycho‐Oncology Cooperative Research Group (PoCoG) School of Psychology The University of Sydney Sydney Australia

## Abstract

**Background:**

Despite improving survival rates, the complexity of post‐operative survivorship for oesophageal cancer patients remains poorly understood. This mixed‐methods review explores quantitative health‐related quality‐of‐life (HRQoL) outcomes and qualitative survivor experiences to provide a comprehensive, person‐centred perspective.

**Methods:**

A convergent synthesis framework was employed to examine patient perspectives on survivorship following oesophageal cancer resection. A systematic search of the literature was undertaken across four databases to identify studies reporting EORTC QLQ‐C30 and QLQ‐OES18 outcomes and qualitative research exploring post‐operative experiences. Quantitative studies underwent random‐effects meta‐analysis to examine HRQoL trajectories from baseline to 5 years post‐operatively. Qualitative studies underwent thematic synthesis following Thomas and Harden's established three‐stage framework.

**Results:**

Fifty‐seven studies were included, consisting of 40 quantitative and 17 qualitative studies. HRQoL followed a triphasic pattern with a marked deterioration at 6 months, partial recovery by 12–36 months, followed by a plateau with persistent symptoms at 5 years. Reflux, eating restriction and fatigue were the most prevalent symptoms following oesophagectomy. Thematic synthesis identified five domains of oesophageal cancer survivorship; digestive disruption, physical impairment, psychosocial impact, navigating healthcare and support systems, and striving for normalcy. Qualitative accounts revealed challenges under‐represented in quantitative measures, including an altered relationship with food, fear of recurrence, as well as identity loss.

**Conclusions:**

Post‐oesophagectomy survivorship is a sustained process of adaptation, not a return to baseline health. Current oncology‐centred care models overlook chronic, multidimensional needs of patients. Integrated survivorship pathways combining nutritional support, rehabilitation, and psychosocial support are essential to restore agency and improve long‐term wellbeing.

## 1 Introduction

Oesophagectomy remains the cornerstone of curative treatment for oesophageal cancer (OC) within the multidisciplinary approach to care [1]. Recent advances in OC care have yielded improvements in patient outcomes, with five‐year relative survival rates now approaching 40%–50% in specialised centres [2]. Whilst this significant progress is a major achievement, it also casts a spotlight on the persistent complexity of post‐cancer resection survivorship.

Functional consequences extend well beyond the immediate postoperative period [3]. Patients face prolonged recovery trajectories extending for years, as they adapt to profound anatomical and physiological changes [4]. OC survivorship is therefore a process of navigating life with a substantially altered body, limited functional capacity, and the long‐term consequences of treatment [5].

Despite the growing population of OC survivors, survivorship care remains fragmented and underdeveloped [6]. Many centres lack standardised follow‐up protocols beyond oncologic surveillance, and patients often report feeling “lost” in the transition from active treatment to survivorship, with inconsistent access to support services [7, 8]. The disconnect between rising survival rates and the day‐to‐day reality of survivors signals the need to refocus attention on quality of life in this population.

To effectively characterise survivorship needs and evaluate care interventions, robust standardised measurements of health‐related quality of life (HRQoL) are therefore essential to capture the unique symptom burden and functional trajectory of OC survivors. The European Organization for Research and Treatment of Cancer Quality of Life Questionnaire‐C30 (EORTC QLQ‐C30) and its oesophageal‐specific module (QLQ‐OES18) represent the gold standard instruments for this assessment of HRQoL [9]. QLQ‐C30 provides scores on global health status as well as various functional and symptom scales, whilst QLQ‐OES18 evaluates symptoms unique to OC.

Quantitative studies have used these instruments to measure recovery over time, yet these aggregate scores inherently lack the contextual depth or nuance in survivor experiences. Qualitative research, however, offers in‐depth perspectives, revealing how survivors interpret their experiences, cope with challenges, and find meaning after surgery [10]. A synthesis of this qualitative evidence is needed to identify common themes and inform person‐centred models of survivorship care. Few reviews have examined the needs of this unique cohort, with most focussing on nutritional rather than psychosocial aspects. This mixed‐methods review aims to integrate longitudinal HRQoL trajectories and qualitative evidence of participants' lived experiences to provide a comprehensive understanding of survivorship after oesophagectomy.

## 2 Methods

This review protocol was registered with the International Prospective Register of Systematic Reviews (PROSPERO) database on 23^rd^ May 2025 (CRD420251020098) [11]. Reporting adhered to the Preferred Reporting Items for Systematic Reviews and Meta‐Analyses (PRISMA) guidelines for systematic reviews and meta‐analyses [12], and the Enhancing Transparency in Reporting the Synthesis of Qualitative Research (ENTREQ) framework for qualitative evidence synthesis [13].

### 2.1 Study Design

This study examined perspectives on survivorship following oesophagectomy for cancer. The integrative review comprised two complementary components: (1) a meta‐analysis of HRQoL outcomes following oesophagectomy to demonstrate the trend from baseline, before oesophagectomy, up to 5 years post‐operatively; and (2) a thematic synthesis of qualitative research to explore major themes in the lived experiences of post‐oesophagectomy survivors. Beyond baseline, HRQoL was assessed at predefined postoperative milestones aligned with established cancer survivorship frameworks [14, 15]: 6 months (acute survivorship), 12 months (transitional survivorship), 36 months (extended survivorship), and 60 months (permanent survivorship).

### 2.2 Search Strategy

A search was performed in CINAHL, MEDLINE, EMBASE, and PsychINFO to identify peer‐reviewed studies exploring HRQoL outcomes and lived experiences of adult survivors who had previously undergone curative‐intent oesophagectomy for cancer. The search strategy incorporated both MeSH terms and free‐text keywords related to “oesophagectomy”, “oesophageal cancer”, “HRQoL”, and “survivorship”. Searches were restricted to English‐language studies published from inception in January 1946 to April 2025. A complete search strategy is provided in (Supporting Information S1: Table S1). Reference lists of all retrieved articles were reviewed to identify any additional relevant studies. Screening of articles and their selection was performed independently by two authors (JS, FA). Disagreements throughout this process were resolved by by consulting a third reviewer.

### 2.3 Eligibility Criteria

For the quantitative component, studies were included if they reported HRQoL data using validated patient‐reported outcome measures, specifically the EORTC QLQ‐C30 and QLQ‐OES18 scales. For the qualitative component, studies were included if they reported on post‐oesophagectomy survivorship experiences and employed recognised qualitative methodologies [16, 17]. All eligible studies were required to meet the following inclusion criteria: (a) report on adults following oesophagectomy for OC; (b) provide original findings from a primary research study; and (c) be published as a full‐text peer‐reviewed article. Studies were omitted based on the following exclusion criteria: (a) focussed solely on pre‐operative phase; (b) described palliative or end‐of‐life experiences; (c) report duplicate data from publications based on the same cohort; (d) data from case reports, editorials, and non‐peer‐reviewed sources.

### 2.4 Data Extraction

A structured data extraction template was developed to capture relevant information including authorship details, study design, participant characteristics, data collection methods, and reported outcomes. Data extraction was performed by two reviewers (JS, TD), with discrepancies resolved through consensus with a third reviewer. Quantitative‐specific data that was extracted involved mean and standard deviation (SD) values of EORTC QLQ‐C30 and EORTC QLQ‐OES18 scores. Qualitative‐specific data that was extracted involved survivor quotes and interpretive insights from the results sections of included studies.

### 2.5 Quantitative Data Synthesis

Meta‐analyses were conducted using random‐effects models to account for between‐study heterogeneity. Effect sizes were reported as mean differences from baseline with 95% confidence intervals (CIs). Primary analyses compared outcomes at baseline, 6, 12, 36, and 60 months postoperatively. Pooled effect estimates for each quality‐of‐life subdomain were presented in forest plots. All statistical analyses were performed using Comprehensive Meta‐Analysis (Biostat, Version 4) and Stata (StataCorp, Version 17).

### 2.6 Qualitative Data Synthesis

Thematic synthesis was conducted following Thomas and Harden's established three‐stage framework [18]. The first stage involved line‐by‐line inductive coding of all relevant survivor quotes and interpretive insights from the results section of included studies into a coding matrix. The second stage involved systematically organising codes into a hierarchical coding framework of descriptive themes, representing recurrent patterns and shared experiences identified across the studies. The frequency and distribution of codes across studies were taken into account during theme development; codes that appeared consistently across multiple studies were given greater interpretive weight than those identified in only a few. The third stage involved grouping the descriptive themes to develop broader analytical themes that offered conceptual insights into post‐oesophagectomy survivorship. Interpretive decisions were reviewed and refined through collaborative discussion, with reflexive practices integrated throughout to challenge assumptions and strengthen the credibility of the findings [19].

### 2.7 Critical Appraisal

The methodological quality of included studies was assessed using the *Joanna Briggs Institute's Critical Appraisal Checklist* for quantitative studies [20], and the *Critical Appraisal Skills Programme (CASP) Checklist* for qualitative studies [21]. Two reviewers independently assessed each study, with disagreements resolved through discussion. Studies were not excluded based solely on quality scores, but these informed the interpretation of findings and confidence in the results.

## 3 Results

### 3.1 Study Selection and Characteristics

**FIGURE 1:**
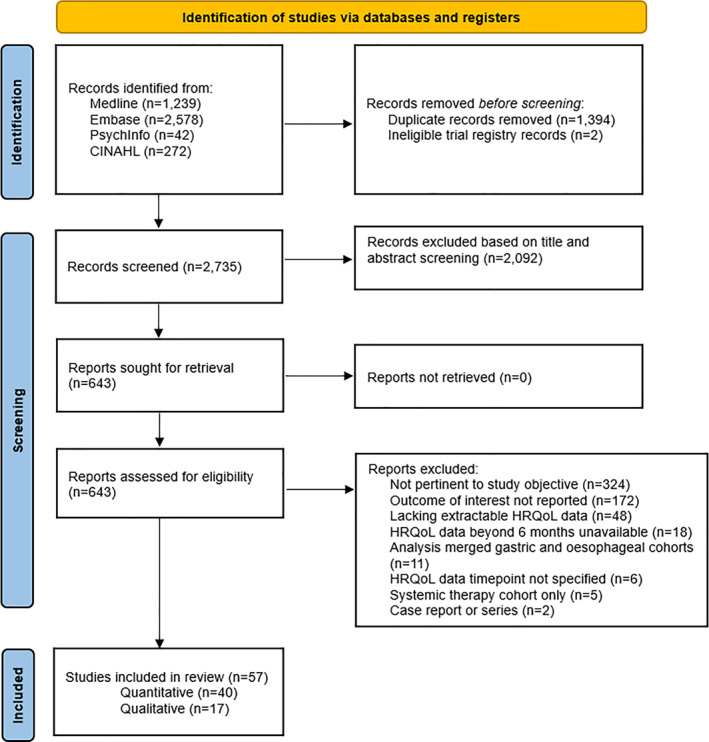
Study selection process adapted from PRISMA.

The search strategy identified 4347 records, from which 683 duplicates were removed. Following title and abstract screening, 643 full‐text articles were assessed for eligibility, resulting in the inclusion of 57 studies in this mixed‐methods systematic review. This included 40 quantitative studies assessing patient‐reported HRQoL outcomes and 17 qualitative studies examining the lived experiences of OC survivors following oesophagectomy. The study selection process is detailed in the PRISMA flow diagram (Figure 1).

The selected studies were published between 1996 and 2025, with general characteristics displayed in Table 1 for quantitative studies and Table 2 for qualitative studies. Participants had mean ages typically ranging from 60 to 67 years, with a marked male predominance, reflecting the epidemiology of oesophageal cancer. The meta‐analyses included studies that assessed HRQoL using validated instruments, the EORTC QLQ‐C30 functional scale and the EORTC QLQ‐OES18 symptom module. The included quantitative studies collectively reported on over 9917 patients focussed on five‐year post‐operative data. Studies pertaining to the thematic synthesis employed methodologies such as in‐depth interviews and focus groups to capture lived experiences following curative‐intent oesophagectomy. These studies represented a broad spectrum of survivorship timelines across 259 survivors, from the early recovery phase to long‐term survival. Thematic analysis was the predominant method of qualitative data analysis.

**TABLE 1:** Characteristics of quantitative studies.

Study	Institution, country	Study type	Aim	Sample size	Cohort characteristics			Timepoint assessed (Months)					EORTC reported	
					Age, year	Gender male, *n* (%)	Procedure, *n* (%)	0	6	12	36	60	QLQ‐C30	QLQ‐OES18
Barbour, 2017 [71]	Princess Alexandra Hospital, Australia	Prospective	Assess HRQoL after thoracoscopic and open oesophagectomy	487	Median 64	414 (85.0%)	Thoracoscopically assisted McKeown oesophagectomy (77.4%); open transthoracic Ivor Lewis oesophagectomy (22.6%)	✔	✔	✔	✔		✔	✔
Bennett, 2022 [72]	Trinity centre for Health sciences, Ireland	Prospective	Investigate HRQoL in oesophageal cancer survivors	66	63.3	55 (83.3%)	Transhiatal oesophagectomy (28.8%); two‐stage oesophagectomy (54.5%); three‐stage oesophagectomy (16.7%)		✔	✔			✔	✔
Cavallin, 2015 [73]	Veneto Institute of oncology IOV IRCCS, Italy	Retrospective	Evaluate the impact of oesophagectomy in elder patients in term of HRQoL	109	Median 61	92 (84.4%)	Ivor‐Lewis oesophagectomy (94.5%); McKeown's oesophagectomy (5.5%)	✔						✔
Chang, 2014 [74]	Chang Gung memorial Hospital, Taiwan	Prospective	Assess HRQoL within 1 and 6 months after surgery and to identify factors predictive of HRQoL within 6 months after esophagectomy in Taiwan	99	55.5	92 (92.9%)	Transthoracic oesophagectomy (56.6%); Transcervical oesophagectomy (43.4%)	✔	✔				✔	✔
Derogar, 2012 [75]	Karolinska Institutet, Sweden	Prospective	Determine HRQoL restoration in 5‐year survivors	117	64	93 (79.5%)	Transthoracic oesophagectomy (82.1%); transhiatal oesophagectomy (17.1%); Transcervical oesophagectomy (0.9%)					✔	✔	✔
Djärv, 2008 [76]	Karolinska Institutet, Sweden	Prospective	Assess HRQoL in patients with surgically cured oesophageal cancer	87		62 (71.3%)	Transthoracic oesophagectomy (78.2%); transhiatal oesophagectomy (21.8%)				✔		✔	✔
Heits, 2018 [77]	University Hospital schleswig‐holstein, Germany	Prospective	Compare HRQoL after endoluminal vacuum therapy with HRQoL of patients without anastomotic leakages	73	63.8	50 (68.5%)	Ivor‐Lewis oesophagectomy (72.6%); McKeown oesophagectomy (21.9%); transhiatal oesophagectomy (5.5%)	✔	✔	✔			✔	
Kataria, 2012 [78]	All India Institute of medical sciences, India	Prospective	Assess HRQoL following transhiatal esophagectomy	30	52	17 (56.7%)	Transhiatal oesophagectomy	✔					✔	✔
Kauppila, 2018 [79]	St Thomas' Hospital, United Kingdom	Prospective	Compar HRQoL of transhiatal oesophagectomy with HRQoL of transthoracic oesophagectomy	146		118 (80.8%)	Transhiatal oesophagectomy (58.9%); transthoracic oesophagectomy (41.1%)		✔	✔			✔	✔
Lagergren, 2007 [80]	University of bristol, United Kingdom	Prospective	Assess HRQoL prospectively in surgically treated patients who survived oesophageal cancer for ≥ 3 postoperative years	47	63	31 (66.0%)	Two‐phase oesophagectomy (89.4%); three‐phase oesophagectomy (4.3%); transhiatal oesophagectomy (6.4%)	✔			✔		✔	✔
Lagergren, 2024 [81]	Karolinska Institutet, Sweden	Prospective	Assess whether complications influence long‐term HRQoL in oesophageal cancer survivors	403	66.7	370 (91.8%)	Minimally invasive oesophagectomy (37.2%); hybrid oesophagectomy (34.2%); open oesophagectomy (28.5%)			✔			✔	✔
Li, 2021 [82]	Fourth Hospital of hebei medical university, China	Retrospective	Compare HRQoL of Chinese patients after minimally invasive esophagectomy with open esophagectomy	104	60.1	64 (61.5%)	Minimally invasive oesophagectomy (72.1%); open oesophagectomy (27.9%)	✔	✔	✔			✔	✔
Lv, 2014 [83]	Qianfoshan Hospital, China	Prospective	Assess the variation in HRQoL of patients receiving definitive chemoradiotherapy or oesophagectomy	50	—	33 (66.0%)	Oesophagectomy not specified	✔	✔				✔	
Maas, 2015 [84]	VU university medical centre, Netherlands	Prospective	Determine whether minimally invasive oesophagectomy has better 1‐year HRQoL than open oesophagectomy	115	Median 62	89 (77.4%)	Minimally invasive oesophagectomy (51.3%); open oesophagectomy (48.7%)	✔		✔			✔	✔
Magon, 2021 [85]	IRCCS policlinico san donato, Italy	Prospective	Describe the trajectories of HRQoL, health literacy, and self‐efficacy in patients with oesophageal cancer at baseline and postoperative	45	63.0	33 (73.3%)	Transhiatal oesophagectomy (6.7%); transthoracic oesophagectomy (77.8%); minimally‐invasive oesophagectomy (13.3%); McKeown oesophagectomy (2.2%)	✔					✔	✔
Mantziari, 2024 [86]	Multicentre (ENSURE), multiple	Prospective	Explore potential sex‐related differences in treatment strategies, operative and oncologic outcomes, and HRQoL after oesophageal cancer treatment with curative intent	3974	64.5	3083 (77.6%)	Open oesophagectomy (67%), hybrid minimally invasive oesophagectomy (16.1%), total minimally invasive oesophagectomy (14.6%), unknown (2.2%)			✔			✔	
Mariette, 2020 [87]	University Lille, France	Prospective	Compare short‐ and long‐term HRQoL following hybrid minimally invasive oesophagectomy and open oesophagectomy	207	61.2	175 (84.5%)	Hybrid minimally invasive oesophagectomy (49.8%); open oesophagectomy (50.2%)	✔					✔	✔
Mohammad, 2016 [88]	Academic medical centre, Netherlands	Retrospective	Measure HRQoL during the course of treatment for oesophageal cancer with curative intent	76	63	59 (77.6%)	Transhiatal oesophagectomy (9.2%); transthoracal oesophagectomy (73.7%); unknown (17.1%)	✔					✔	✔
Nafteux, 2013 [89]	University Hospitals Leuven, Belgium	Prospective	Evaluate baseline HRQoL factors that influence short‐term outcome after oesophagectomy and the effects of postoperative length of hospital stay on postoperative HRQoL	455	63.1	372 (81.8%)	Transthoracic cervical anastomosis (73.2%); transthoracic intrathoracic anastomosis (6.4%); transthoracic total gastrectomy (4.6%); minimally invasive oesophagectomy (15.8%)	✔		✔			✔	✔
Nath, 2023 [90]	Thanjavur medical college, India	Prospective	Determine the short‐term morbidity and HRQoL of patients with minimally invasive esophagectomy	15	50.4	10 (66.7%)	Thoraco‐laparoscopic oesophagectomy (13.3%); hybrid oesophagectomy (86.7%)			✔			✔	✔
Oberhoff, 2024 [91]	Uniklinik Aachen, Germany	Prospective	Evaluate nutritional status and associated symptoms 6 months after oesophagectomy	24	64.3	20 (83.3%)	Minimal invasive oesophagectomy	✔	✔				✔	✔
Parameswaran, 2010 [92]	University of bristol, United Kingdom	Prospective	Assess the impact of minimally invasive oesophagectomy on HRQL	62	Median 67	56 (90.3%)	Minimal invasive oesophagectomy	✔	✔	✔			✔	✔
Park, 2021 [93]	Yonsei university college of medicine, South Korea	Retrospective	Evaluate HRQoL after reconstruction by either substernal or posterior mediastinal routes in the McKeown procedure	378	63.4	345 (91.3%)	McKeown procedure — substernal route reconstruction (73.5%); posterior mediastinal route reconstruction (26.5%)	✔	✔	✔			✔	✔
Ramakrishnaiah, 2014 [94]	Jawaharlal Institute of postgraduate medical education and research, India	Prospective	Assess HRQoL following oesophagectomy	55	52.6	33 (60.0%)	Transhiatal oesophagectomy (67.3%); transthoracic oesophagectomy (32.7%)	✔	✔	✔			✔	✔
Reynolds, 2006 [95]	St James's Hospital and Trinity college Dublin, Ireland	Prospective	Evaluate HRQoL for up to 1 year after surgery in patients treated with curative intent by surgery alone or a multimodal approach	202	Median 64	—	Two‐stage oesophagectomy (57.9%); three‐stage oesophagectomy (27.2%); transhiatal oesophagectomy (2.0%); left thoracoabdominal oesophagectomy (6.9%), unknown (5.9%)	✔	✔	✔			✔	
Schandl, 2016 [96]	Karolinska Institutet, Sweden	Prospective	Determine whether oesophageal cancer survivors recover in HRQoL within 10 years of surgery	92	—	73 (79.3%)	Transthoracic oesophagectomy (82.6%), transhiatal oesophagectomy (15.2%), Transcervical oesophagectomy (2.2%)					✔	✔	✔
Sunde, 2019 [97]	Karolinska Institutet, Sweden	Prospective	Address short‐ and long‐term HRQoL amongst patients randomised in the NeoRes trial	154	*Range 37–75*	26 (16.9%)	Oesophagectomy not specified	✔					✔	✔
Tatematsu, 2013 [98]	Kyoto university, Japan	Prospective	Evaluate the impact of oesophagectomy on physical fitness and HRQOL	30	63.6	25 (83.3%)	Oesophagectomy not specified	✔					✔	
Van meerten, 2008 [99]	University medical centre, Netherlands	Prospective	Evaluate HRQoL up to 1 year after surgery in patients treated with curative intent using neoadjuvant non‐platinum‐based chemoradiotherapy followed by oesophagectomy	54	Median 59	90.7%	Oesophagectomy not specified	✔	✔	✔			✔	✔
Viklund, 2006 [100]	Karolinska Institutet, Sweden	Prospective	Assess HRQoL and symptoms after oesophageal cancer surgery	282	Median 67	79.4%	Transthoracic oesophagectomy (82.6%); transhiatal oesophagectomy (17.4%)		✔				✔	✔
Wang, 2010 [101]	Fudan university, China	Retrospective	Compare the short‐term HRQoL in patients after subtotal oesophagectomy via VATS versus OE	29	58.2	Male 19 (65.5%)	Open oesophagectomy	✔	✔				✔	✔
Wang, 2010 [102]	Fudan university, China	Retrospective	Compare the short‐term HRQoL between the two different routes of gastric tube reconstruction after minimally invasive esophagectomy	97	61.1	Male 62 (63.9%)	Minimally invasive oesophagectomy ‐ retrosternal route (49.5%); prevertebral route (50.5%)	✔	✔				✔	
Wang, 2015 [103]	Fudan university, China	Retrospective	Compare postoperative HRQoL in a matched population of patients undergoing minimally invasive oesophagectomy with a control open oesophagectomy group	888	Median 56	720 (81.1%)	Minimally invasive oesophagectomy (50%); open oesophagectomy (50%)	✔	✔	✔			✔	
Williams, 2022 [104]	University of Michigan, USA	Prospective	Compare long‐term HRQoL for patients undergoing transhiatal robotic assisted minimally invasive oesophagectomy or open transhiatal esophagectomy for clinical stage I to III oesophageal cancer	309	65.7	276 (89.3%)	Open transhiatal oesophagectomy (68.6%); transhiatal robotic assisted oesophagectomy 97 (31.4%)	✔	✔	✔			✔	✔
Yang, 2022 [105]	Henan cancer Hospital, China	Retrospective	Explore the effects of early, quantified, modified oral feeding protocol for patients after oesophagectomy on HRQoL	315	63.1	212 (67.3%)	Minimally invasive esophagectomy	✔					✔	✔
Zapletal, 2014 [106]	Helios dr. Horst schmidt Hospital wiesbaden, Germany	Prospective	Compare HRQL between these transthoracic oesophagectomy and modified merendino resection	77	62	60 (77.9%)	Transthoracic oesophagectomy (67.5%); modified merendino resection (32.5%)	✔		✔			✔	✔
Zeng, 2017 [107]	Medical college of zhejiang university, China	Prospective	Characterise the effect of home enteral nutrition on the HRQoL of patients who underwent Ivor Lewis oesophagectomy	60	60.5	46 (76.7%)	Ivor‐Lewis oesophagectomy		✔				✔	
Zhang, 2015 [108]	First Affiliated Hospital of chongqing medical university, China	Prospective	Compare the effect of narrow gastric tube reconstruction and whole‐stomach reconstruction on the long‐term HRQoL in patients after oesophagectomy	104	60.1	78 (75.0%)	Oesophagectomy not specified		✔	✔	✔	✔	✔	✔

*Note:* Data are displayed as mean unless stated otherwise.

**TABLE 2:** Characteristics of qualitative studies.

Author	Institution, country	Aim	Data collection/Research design	Cohort characteristics	Stage of treatment	Main themes
Bennett, 2020 [29]	Trinity centre for Health sciences, Ireland	Examine patient experiences of oesophageal cancer diagnosis, treatment, and recovery	Focus‐group interviews; qualitative content analysis	18 post‐oesophagectomy cancer survivors; range of 8–62 months post‐operative; mean age 67.8; gender 14 M:4 F	Within 5 years post‐operatively	Receiving a diagnosis of oesophageal cancer; navigating treatment of oesophageal cancer; early stages of recovery after treatment; later stages of recovery after treatment
Bull, 2019 [33]	Flinders centre for Innovation in cancer, Australia	Explore views of patients, carers, and HCPs on rehabilitation	Semi‐structured interviews; framework analysis	15 post‐oesophagectomy cancer survivors; mean age 69.6; gender 13 M:2 F	Mixed phases	Getting back to normal
Carey, 2013 [28]	Royal prince Alfred Hospital, Australia	Explore the long‐term impact of different types of major upper gastrointestinal surgeries on people's relationship with food.	Semi‐structured Interviews; thematic analysis	7 post‐oesophagectomy cancer survivors with roux‐en‐y reconstruction; unknown age and gender distribution	6 months to 10 years post‐operatively	Physical symptoms; weight; emotional and social impact; control/Loss; supports; resignation
Cass, 2024 [23]	MD Anderson cancer centre, USA	Explore symptom burden of patients throughout their perioperative course after upper gastrointestinal cancers.	Semi‐structured interviews; symptom inventory development	5 post‐oesophagectomy cancer survivors; unknown age and gender distribution	Mixed phases	Symptom burden; eating changes; coping
Czornik, 2024 [32]	University of freiburg, Germany	Explore patient preferences and needs regarding treatment options after neoadjuvant therapy.	Semi‐structured interviews; qualitative content analysis	5 post‐oesophagectomy cancer survivors; mean of 91.2 days post‐operative; mean age 59.2; gender 4 M:1 F	Mean 3 months post‐operatively	Negative aspects of surgery; positive aspects of surgery; information needs; decision‐making
Gillman, 2024 [25]	St james' Hospital, Ireland	Understand the long‐term impact of aerodigestive symptoms on quality of life in adults post‐oesophagectomy.	Semi‐structured interviews; thematic analysis	40 post‐oesophagectomy cancer survivors; mean of 51.7 months post‐operative; mean age 66.0 years; gender 30 M:10 F	1–11 years post‐operatively	Isolation; fear of complications/recurrence; altered work capacity; avoidance of social eating
Graham‐wisener, 2019 [35]	Belfast Health and social care Trust, Ireland	Explore views on psychological distress, support needs, and preferred interventions in oesophageal cancer care.	Semi‐structured interviews; directed content analysis	3 post‐oesophagectomy cancer survivors; mean of 45 months post‐operative; gender 2 M:1 F	Mean 45 months post‐operatively	Heightened distress at diagnosis and post‐discharge; inadequate support provision; barriers to engagement; need for tailored support
Hellstadius, 2018 [24]	Regional cancer centre, Sweden	Describe how patients emotionally adapt during the first 6 months after diagnosis and surgery for oesophageal cancer.	Semi‐structured interview approach; qualitative content analysis	3 post‐oesophagectomy cancer survivors; mean of 45 months post‐operative; unknown age; gender 8 M:2 F	Within 6 months post‐operatively	Experiencing a crisis reaction
Heramb‐Aamot, 2024 [26]	Oslo University Hospital, Norway	Explore patients experience of everyday life and recovery after esophagectomy.	In‐depth and semi‐structured interviews; Phenomenology	6 post‐oesophagectomy cancer survivors; approximately 6–8 months post‐operative; age range 40–67; gender 6 M:0 F	6–8 months post‐operatively	Eating difficulties; living with uncertainty; relationship; work; identity; social activities
Jaromahum, 2010 [109]	Morristown memorial Hospital, USA	Understand patients' lived experiences of eating after oesophagectomy.	Semi‐structured Interviews; Phenomenology	7 post‐oesophagectomy cancer survivors; early post‐operative period; mean age 62; gender 5 M:2 F	During the acute post‐operative hospitalisation	Gastrointestinal feelings; fear; positive feelings towards eating
Li, 2023 [7]	Sun Yat‐sen university, China	Understand the supportive care needs of patients after esophagectomy.	Semi‐structured interviews; thematic analysis	20 post‐oesophagectomy cancer survivors; mean of 5.56 months post‐operative; mean age 63.5; gender 14 M:6 F	2–5 years post‐operatively	Symptom management needs; dietary/nutritional needs; psychosocial adjustment needs; social support needs
Malmström, 2013 [37]	Skåne university Hospital, Sweden	Explore patients' long‐term experiences of supportive care after oesophagectomy or oesophagogastrectomy for cancer.	Semi‐ structured focus group interviews; qualitative content analysis	17 post‐oesophagectomy cancer survivors; range of 2–5 years post‐operative; age range 46–89; gender 14 M:3 F	2–5 years post‐operatively	Need for guidance in new life situation
McCorry, 2009 [30]	Queen's university belfast, UK	Explore the emotional, cognitive, and social experiences of oesophageal cancer survivors and their carers after surgery.	Focus‐group Interviews; qualitative content analysis	12 post‐oesophagectomy cancer survivors; range of 7–204 months post‐operative; age range 56–85; gender 9 M:3 F	7 months to 17 years post‐operatively	Coping with a death sentence; adjusting to and accepting an altered self; unique benefits of peer support
Nielsen, 2021 [34]	Karolinska Institutet, Sweden	Explore the advice oesophageal cancer surgery patients would give to future patients based on their lived experiences.	Semi‐structured Interview; qualitative content analysis	63 post‐oesophagectomy cancer survivors; 1 Year post‐operative; mean age 68.3; unknown gender distribution	1 year post‐operatively	Health promoting advice; acknowledging the new situation; embracing support from others
O'Neill, 2021 [31]	Trinity college Dublin, Ireland	Explore patients' perspectives on physical recovery and rehabilitation needs in the first 6 months after surgery.	Semi‐structured Interview; qualitative content analysis	18 post‐oesophagectomy cancer survivors; approximately 10 months post‐operative; unknown age of cohort; unknown gender distribution	4 weeks to 6 months post‐operatively	Recovery challenges; return to activity; role resumption; rehab recommendations
Sadeghi, 2025 [36]	Trinity college Dublin, Ireland	Explore nutrition challenges and unmet care needs in oesophago‐gastric cancer survivors.	Semi‐structured interviews; thematic analysis	8 post‐oesophagectomy cancer survivors; mean of 62.8 months post‐operative; mean age 61; gender 7 M:1 F	2–10 years post‐operatively (long‐term)	Nutrition‐related challenges and complications; experiences with dietetic services; coping strategies
Sjeltoft, 2020 [27]	Copenhagen University Hospital, Denmark	Explore patients' lived experiences of eating and daily life during the first year after oesophageal cancer surgery.	Semi‐structured interview; phenomenology	13 post‐oesophagectomy cancer survivors; 1 Year post‐operative; mean age 65; gender 7 M:6 F	Within a year post‐operatively	Adjusting to a different anatomy; changed body; feeling different; nutritional jungle

Quantitative studies were generally of reasonable quality, with only one rated as high risk‐of‐bias (Supporting Information S1: Table S2). Sensitivity analysis excluding the high risk‐of‐bias study demonstrated minimal impact on pooled estimates and did not alter the direction or magnitude of the findings (Supporting Information S1: Tables S3 and S4). Qualitative studies demonstrated similarly robust methodological quality, with the majority judged high quality on CASP appraisal and only one study classified as moderate quality (Supporting Information S1: Table S5).

### 3.2 Aggregated Health‐Related Quality of Life Outcomes

**FIGURE 2:**
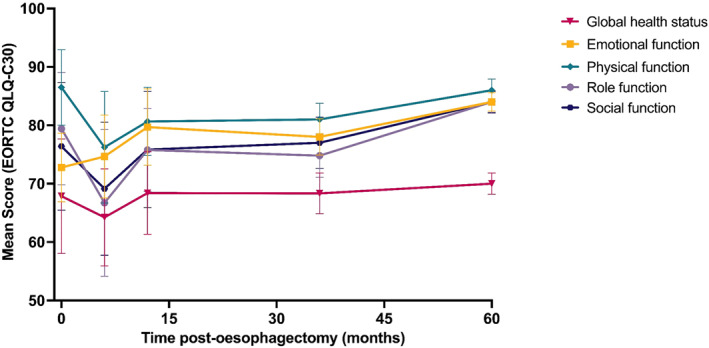
Longitudinal changes in EORTC QLQ‐C30 global health status and functional scales after oesophagectomy.

Functional status trajectories: Analysis of functional domains over 5 years revealed a consistent pattern of acute deterioration followed by partial recovery (Figure 2). Physical function significantly declined with a 10.3‐point decrease during the initial 6 months after surgery, followed by gradual recovery and approaching baseline level at 5 years postoperatively. Role function dramatically deteriorated in the first 6 months (falling from 79.4 to 66.7) though recovered well over the next 3‐year post‐operatively, and ultimately surpassed baseline scores. Social function also followed the pattern of initial decline (76.4–69.1) with subsequent recovery to 75.8 by 12 months. Emotional function, however, demonstrated a unique trend in which scores progressively improved following oesophagectomy throughout the follow‐up period (Supporting Information S1: Table S6).

**FIGURE 3:**
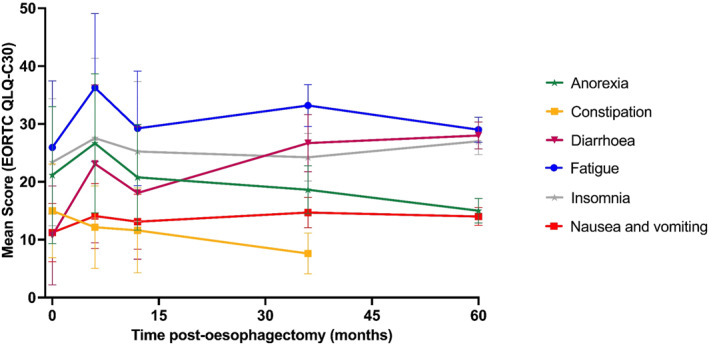
Longitudinal changes in EORTC QLQ‐C30 symptom scales after oesophagectomy.

General symptom burden: Symptom trajectories inversely mirrored functional decline, as shown in Figure 3, with marked increases at 6 months. During this early post‐operative phase, patients experienced substantially elevated symptom scores compared to normative reference values, which represent standardised average scores from the general healthy population as established by the EORTC Quality of Life Group [22]. The most burdensome symptoms were fatigue (36.3 vs. 29.5), insomnia (27.5 vs. 26.6), anorexia (26.6 vs. 10.0), and dyspnoea (26.5 vs. 15.9). Whilst most symptoms showed partial improvement after 12 months post‐operatively, gastrointestinal symptoms exhibited divergent patterns. The presence of nausea and vomiting, as well as diarrhoea, demonstrated ongoing fluctuations throughout the recovery period, with 5‐year post‐operative scores remaining 8.1 and 18.5 points above normative reference ranges, respectively.

**FIGURE 4:**
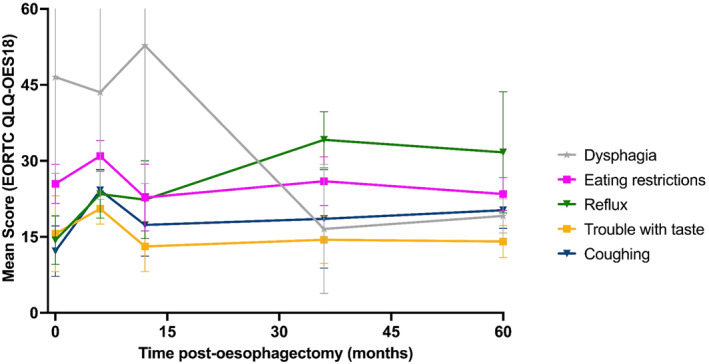
Longitudinal changes in EORTC QLQ‐OES18 oesophageal symptom scores after oesophagectomy.

Oesophageal‐specific symptom burden: Oesophageal‐specific symptoms highlighted the central therapeutic trade‐off of surgery. Dysphagia, the predominant preoperative symptom with a baseline score of 46.5, decreased significantly to 16.6 at 36 months, maintaining low levels at 19.1 through 60 months (Figure 4). Other oesophageal symptoms such as eating restriction and odynophagia showed little sustained improvement, often remaining stable or even worsening throughout survivorship. Reflux progressively increased from 14.3 at baseline to 34.2 at 36 months, plateauing at approximately 32.0 through 60 months. This increase in reflux corresponded with progressive worsening of coughing (12.2–20.3).

Supporting Information S1: Figures S2 and S3 show forest plot meta‐analyses at 12 and 60 months post‐operatively, representing key recovery milestones along the trajectory from postoperative recovery to long‐term survivorship. HRQoL outcomes at the 5‐year postoperative time point were reported by only two studies, resulting in a limited evidence base informing long‐term estimate. Assessment of potential publication bias is shown in Supporting Information S1: Figure S3, with no clear evidence of substantial small‐study effects.

### 3.3 Thematic Synthesis

**FIGURE 5:**
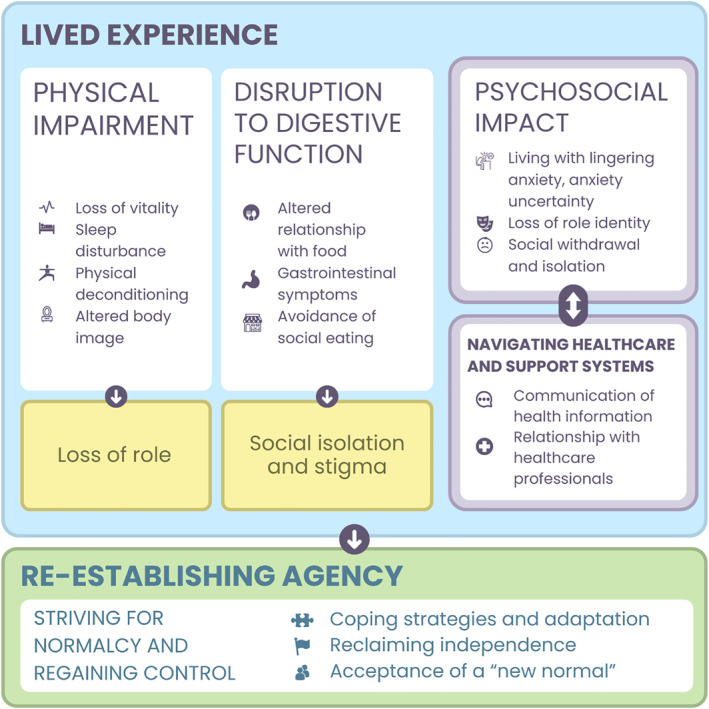
Conceptual model of the survivorship journey after oesophagectomy.

Thematic synthesis yielded five overarching analytical themes of post‐oesophagectomy survivorship, each composed of interrelated descriptive subthemes as shown in Figure 5; (1) disruption to digestive function; (2) physical impairment; (3) psychosocial impact; (4) navigating healthcare and support systems; and (5) striving for normalcy and regaining control. These themes reflect evolving challenges and adaptive strategies of OC survivors. Whilst digestive disruption and physical impairment are closely related, they were maintained as distinct themes to preserve the specificity of the survivor experience. Table 3 provides an overview of each study's contribution to the identified themes, whilst illustrative participant quotations for each analytical and descriptive theme are presented in the Supporting Information S1: Table S7.

**TABLE 3:** Overview of the contribution of each study to the themes.

Included studies		Bennett, 2020 [29]	Bull, 2019 [33]	Carey, 2013 [28]	Cass, 2024 [23]	Czornik, 2024 [32]	Gillman, 2024 [25]	Graham‐Wisener, 2019 [35]	Hellstadius, 2018 [24]	Heramb‐Aamot, 2024 [26]	Jaromahum, 2010 [109]	Li, 2023 [7]	Malmström, 2013 [37]	McCorry, 2009 [30]	Nielsen, 2021 [34]	O'Neill, 2021 [31]	Sadeghi, 2025 [36]	Sjeltoft, 2020 [27]
Quality of evidence (score)		High (19)	High (19)	High (19)	High (20)	High (20)	High (20)	High (18)	High (20)	High (20)	Moderate (16)	High (20)	High (19)	High (19)	High (20)	High (20)	High (20)	High (18)
Analytical themes	Descriptive themes																	
Disruption to digestive function	Altered relationship with food				✔		✔		✔	✔	✔	✔				✔	✔	✔
	Gastrointestinal symptoms	✔		✔	✔		✔				✔	✔		✔	✔	✔	✔	✔
	Avoidance of social eating					✔	✔			✔		✔		✔			✔	
Physical impairment	Loss of vitality				✔	✔			✔							✔		
	Sleep disturbance									✔		✔						
	Physical deconditioning	✔							✔	✔		✔				✔		
	Altered body image			✔		✔			✔	✔						✔		
Psychosocial impact	Living with lingering anxiety and uncertainty	✔	✔		✔		✔		✔	✔		✔		✔		✔		
	Loss of role identity	✔	✔				✔		✔			✔				✔		
	Social withdrawal and isolation	✔					✔	✔	✔	✔		✔	✔			✔		✔
Navigating healthcare and support systems	Communication of health information	✔		✔		✔	✔	✔		✔		✔	✔			✔		
	Relationship with healthcare professionals		✔	✔		✔	✔	✔					✔	✔	✔			
	Importance of psychosocial and peer support			✔			✔	✔				✔	✔	✔		✔	✔	✔
Striving for normalcy and regaining control	Coping strategies and adaption	✔	✔				✔		✔					✔	✔	✔	✔	✔
	Reclaiming independence									✔	✔				✔	✔		
	Acceptance of a 'new normal'								✔	✔					✔	✔	✔	

### 3.4 Disruption to Digestive Function

The first analytical theme captured the profound impact of altered gastrointestinal anatomy and physiology on survivors' dietary behaviours and daily routines.

#### 3.4.1 Altered Relationship With Food

Survivors described a distressing transformation in their relationship with food, which was no longer pleasurable but effortful and mechanical [23, 24]. Many compared meals to “taking an antibiotic”, or eating purely out of necessity, “I'm eating because I have to”, “It doesn't feel like food” [25, 26]. These statements reflect a broader sense of food aversion and reduced enjoyment from “force consumption” [23, 24, 26, 27].

#### 3.4.2 Gastrointestinal Symptoms

A wide spectrum of persistent and unpredictable gastrointestinal symptoms was reported [25, 28], often described as intrusive and exhausting in daily life. The physical consequences of eating were frequently severe, as recounted, “I had to learn to eat more slowly [to avoid]…. Horrendous, terrible pain” [29]. Fear of sudden diarrhoea or post‐meal vomiting often led to food avoidance or hypervigilance around mealtimes, with survivors constantly “checking that [they] don't eat too much” to prevent adverse symptoms [27, 29]. This persistent symptom burden interfered not only with nutritional intake but also with emotional wellbeing, reinforcing feelings of vulnerability and frustration throughout the survivorship period.

#### 3.4.3 Avoidance of Social Eating

The social ramifications of digestive dysfunction were also profound, leading to the avoidance of shared meals, especially in publick or family settings [26, 30]. This withdrawal was attributed to a fear of embarrassment from gastrointestinal symptoms or the inability to tolerate a full meal, causing survivors to decline invitations or feel conspicuous for ordering small portions [26]. Restaurant experiences appeared to be inherently stressful, with participants noting they “hate going out for a meal [and]… just stopped going out” [25]. These experiences disrupted important social rituals and amplified feelings of difference and exclusion.

### 3.5 Physical Impairment

This theme reflects the profound physical challenges that patients experienced following oesophagectomy. Survivors described persistent fatigue, muscle weakness, and sleep disturbances that created a pervasive sense of bodily vulnerability and significantly disrupted their daily functioning and prolonged recovery.

#### 3.5.1 Loss of Vitality

Loss of vitality was a central concern, described as feeling “total knackered all over” and persisting for months or years, distinct from typical tiredness [29, 31]. The fatigue was often described as multi‐dimensional, simultaneously weighing on “both psychological and physical [capacities] in some way” [24]. Even basic activities were exhausting, with some survivors reporting the need to “take a nap… after each meal” [32].

#### 3.5.2 Physical Deconditioning

Muscle weakness, with the subsequent reduction in mobility and difficulty performing everyday tasks emerged as an enduring consequence of treatment [7, 33]. One participant reflected, “I've got no energy in my body, my muscles aren't working either” [26]. Beyond general weakness, participants described muscle atrophy and impaired coordination, all of which hindered physical recovery. Several survivors acknowledged that they were “conscious of the fact that [they've] lost a lot of muscle” [31]. Physical deconditioning represented not merely a temporary setback but a sustained challenge that persisted months after treatment completion, leading many patients to recognise the need for further postoperative physiotherapy and targeted rehabilitation [7, 33].

#### 3.5.3 Sleep Disturbance

Sleep was commonly disrupted by reflux or psychological distress, further exacerbating functional decline [7]. Survivors emphasised how their persistent symptoms interfered with rest, “I can't sleep! It has been a long time… experience also affected sleep quality with one participant describing being kept awake by “lots of thoughts swirling around in [their] head… at night” [26].

#### 3.5.4 Altered Body Image

Changes in body image, commonly from either significant weight loss or surgical scars, were associated with anxiety, shame, and a sense of disempowerment. Survivors described feeling “too skinny” or “disgusting” [24, 28], leading to social self‐consciousness and a disruption of their identity. The statement, “I worry about my weight… it looks terrible,” typifies the distress and body image concerns repeatedly described by participants [24]. The physical changes extended beyond weight, with survivors disclosing “embarrassment… with the scars and with the stitching” [31]. Survivors expressed feeling “ashamed of [their] body today”, with these changes deeply tied to identity disruption and a sense of embodied vulnerability [24].

### 3.6 Psychosocial Impact

The emotional toll of recovery emerged as a strong theme throughout, characterised by profound psychological distress and disruptions to social roles and relationships.

#### 3.6.1 Living With Lingering Anxiety and Uncertainty

Living with lingering anxiety and uncertainty was a defining feature of post‐operative survivorship. Survivors reported persistent emotional turbulence, shaped by the continued state of vigilance towards the possibility of recurrences and complications [23, 30, 34]. Phrases such as “You don't know when you'll be getting measured for the coffin,” and the constant thought “It's in the back of my mind that it will suddenly come back one day” captured the constant mental burden [23, 26, 34]. Although anxiety and depressive symptoms were noted to ease gradually with time, uncertainty or “fear of the unknown” remained a defining feature of recovery [23].

#### 3.6.2 Loss of Role Identity

Many struggled with a loss of identity that manifested as both an inability to resume previous social or occupational roles, as well as a broader disconnection from their normal life [29, 30]. The inability to fulfil previous roles was deeply distressing, with many patients in the early recovery phase resisting help with daily activities, asserting, “It's my responsibility… I want to do it” [29]. These survivors simultaneously felt marginalised when excluded from performing their normal role [29]. Work capacity was frequently undermined by physical limitations and embarrassment, with many describing return to work as a “significant problem” due to persistent aerodigestive symptoms and fatigue [25]. Survivors frequently described workplace challenges, including the difficulty managing meals around rigid schedules, prolonged post‐meal pain, and the constant need for ready toilet access. Open‐plan offices were often seen as incompatible due to symptoms such as belching and reflux, whilst the opportunity to work from home during COVID‐19 pandemic was described as “a blessing in disguise” [25]. This role disruption was compounded by a profound sense of detachment from normal life, with survivors describing themselves during recovery as being in a “different world” or “complete limbo,” which further hindered their engagement in previously routine activities [30].

#### 3.6.3 Social Withdrawal and Isolation

Social withdrawal was a common and multifaceted experience, driven by a combination of physical impairments, communication difficulties, and emotional fatigue [7, 35]. Survivors frequently described feeling “totally alone” during recovery [25], and many reported deliberately retreating from social engagement, particularly during the early, more vulnerable phases after surgery, citing the “extra effort” required to interact as overwhelming [30]. Physical and communication barriers, such as hoarse speech or the presence of a feeding tube, also contributed to a sense of social isolation, with one survivor sharing, “I dare not go out… It must be a bit awkward to meet people…” [7]. This was especially pronounced for older or more vulnerable survivors, who faced additional logistical challenges, making it “difficult for them to attend any type of appointment” and leaving them feeling increasingly marginalised from their communities and support networks [35].

### 3.7 Navigating Healthcare and Support Systems

Survivors frequently highlighted the complex challenges of engaging with healthcare and support services during recovery. Although some reported positive and trusting relationships with their clinical teams, many described gaps in communication, inconsistent follow‐up, and limited access to psychosocial support.

#### 3.7.1 Communication of Health Information

Communication of health information was often inadequate or unclear, particularly post‐discharge [28, 33]. Patients struggled with medical terminology, referring to them as “foreign words,” and felt overwhelmed by complex information [32, 36]. The need for more tailored information was evident [29], with suggestions for “a one‐leaflet‐fits‐all” approach to be replaced by more practical and personalised guidance, potentially through short videos or a centralised accessible website [36]. Where formal guidance was lacking, many turned to the internet for answers, though this often heightened anxiety when confronted with poor survival data, “If you go on the internet… you didn't see very comforting statistics there… It was frightening” [29].

#### 3.7.2 Relationship With Healthcare Professionals

Trust and continuity with healthcare professionals varied significantly, with gaps in understanding and follow‐up being common concerns [25, 32]. Some participants did express confidence in their surgical teams, expressing that “they know what they're doing” [32]. Some survivors expressed frustration that general healthcare providers lacked insight into post‐operative challenges, claiming “they seem to know absolutely nothing about it” [33], reflecting the systemic gaps in continuity of care beyond specialist centres. Time constraints during clinical encounters was also problematic with some survivors requesting “a little more time for the patients,” and others acknowledged “surgeons don't have time” [32]. Participants expressed concern about the lack of continued multidisciplinary care, highlighting the need for prolonged access to dietitians and specialised nursing support, as many felt abruptly left to manage on their own during the later stages of recovery [36].

#### 3.7.3 Importance of Psychosocial and Peer Support

Access to psychosocial support was inconsistent, leading to feelings of abandonment [29, 37], as expressed in the following quotes, “There's nothing really in terms of support,” and “I never felt I had any real support after that [the first 6 months]… I felt very isolated, on my own” [36]. Survivors advocated for the need for continued support from either a counsellor or a survivor group [30]. Peer support was uniquely valued for its validating and empowering role, providing emotional reassurance and practical strategies, particularly in the absence of professional continuity. Survivors valued connexion with others who understood their experience, claiming that support groups “makes you feel normal” [30]. Access to such groups was frequently limited [25], as survivors were rarely linked in with each other, prompting calls for “more support groups” or a “WhatsApp group” [29, 36].

### 3.8 Striving for Normalcy and Regaining Control

This theme embodies survivors' active efforts to rebuild structure and reclaim agency amidst major physical and psychosocial disruption.

#### 3.8.1 Coping Strategies and Adaptation

Survivors adopted various strategies to manage the ongoing challenges of recovery [30, 34]. Mental resilience was fostered by setting manageable daily goals, “[taking] it a day at a time,” and reframing setbacks as temporary [25]. Routine‐building helped sustain momentum for patients to continue on days even when motivation was low [30]. Many found emotional grounding through maintaining a sense of purpose through hobbies, faith, or social connections. Physical activity emerged as a particularly powerful coping mechanism in this way, often described as energising and preferable to “just laying down and waiting [where] nothing really happens” [34].

#### 3.8.2 Reclaiming Independence

Regaining a sense of autonomy through managing routine activities of daily living without constant assistance was seen as a pivotal milestone. Many survivors also described reclaiming independence and feeling “normal again” after returning to work, resuming driving, or re‐engaging socially [30, 31]. Whilst the transition from dependence to independence was often gradual and marked by occasional setbacks, these small everyday tasks carried deep symbolic value for survivors [31]. Reclaiming everyday responsibilities signified not only physical improvement but also emotional progress and the reconstruction of normalcy and a post‐cancer identity.

#### 3.8.3 Acceptance of a 'New Normal'

Some survivors reconstructed their identity through acceptance and personal growth over time, redefining what “normal” meant rather than attempting to return to their pre‐illness state [24, 26]. Acceptance was not as passive resignation, but as active resilience, and many eventually embraced “a new way of living” [24]. Survivors gradually “learned to work with it, [and] not fight against” their altered bodies, incorporating changes into a revised sense of self [30]. This reconstruction involved letting go of former expectations, adjusting personal goals, and embracing routines that accommodated their changed capacities. Many survivors found this process empowering, suggesting that “it gives life experience”, offering opportunities to reassess priorities and develop renewed appreciation for life [34].

## 4 Discussion

This review demonstrates that recovery after oesophagectomy is not a linear return to pre‐illness functioning but rather a complex long‐term process of adaptation. The distinct triphasic HRQoL pattern following oesophagectomy comprised an early acute decline in function with a corresponding peak in symptom burden. The pronounced 6‐month deterioration has clear biological underpinnings, coinciding with the cumulative impact of major anatomical reconstruction and treatment‐related toxicity [38, 39]. Subsequent partial functional recovery is then observed by 12–36 months, a reflection of ongoing adaptation, followed by a long‐term plateau marked by persistent digestive, physical and psychosocial sequelae, consistent with the qualitative insights of lived experiences. This highlights gaps in current survivorship care models.

Whilst the other broader analytical themes resonate across all cancer survivorship populations, digestive disruption represents a particularly unique challenge for oesophagectomy survivors. Unlike other cancer cohorts who may selectively avoid triggers, OC survivors must confront their altered physiology multiple times daily at every meal, making this fundamental necessity a source of anxiety and potential embarrassment [40]. Qualitative syntheses consistently describe persistent gastrointestinal symptoms as lifelong, shaping not only nutrition but also social participation and identity [40]. Survivors emphasised the need for proactive, continuous dietetic input to help manage dysphagia, anorexia and reflux, yet many report limited access to specialist support. This mismatch between need and provision highlights the necessity for integrated nutritional pathways and interdisciplinary care, extending beyond hospital discharge and into survivorship. The recent *REsolution of Symptoms afTer Oesophago‐gastric cancer REsection delphi* (RESTOREd) consensus similarly echoed this need, calling for integrated multidisciplinary care involving gastroenterologists and dietitians to better identify and address these persistent functional complications [41]. A significant subset of survivors after oesophagectomy do also experience more severe functional gastrointestinal problems such as delayed gastric conduit emptying (DGCE), dumping syndrome, refractory gastroesophageal reflux, and rumination syndrome [42]. DGCE is especially prevalent, affecting up to 50% of survivors and causing stasis of food that manifests as nausea, regurgitation, and malnutrition, collectively imposing significant quality‐of‐life impairments [43]. Although not all survivors experience such intense functional complications, those who do bear a disproportionate symptom burden, reflected in qualitative accounts of unpredictable and intrusive gastrointestinal symptoms that dominate daily life [44]. Taken together, these findings provide a patient‐centred rationale for the development of an oesophageal cancer‐specific nutritional survivorship pathway that integrates screening for functional complications and sustained dietetic care with psychosocial support beyond the immediate postoperative period.

Whilst physical function approached baseline levels by 12 months post‐operatively, this apparent recovery may reflect reconceptualisation of wellbeing and adaptation to new limitations, rather than a true restoration of pre‐illness capacity [45]. This aligns with the *response shift theory*, whereby survivors recalibrate what they consider acceptable function after major illness, potentially obscuring persistent impairment in self‐reported measures [46, 47]. At long‐term follow‐up (> 3–5 years), fatigue and insomnia improved compared with the acute post‐operative phase, though persisted above normative levels, reflecting a chronically altered baseline. This apparent stabilisation of HRQoL masks the continued salience of physical impairment in survivors' daily lives, with qualitative accounts describing pervasive tiredness, reduced stamina, and sleep disruption when these themes were examined in greater depth [48].

An important discrepancy also emerged between quantitative psychosocial scores and qualitative experiences of survivors. Emotional and social function scales suggested improvement over time, yet lived accounts revealed persistent fear of recurrence, loss of identity, and withdrawal from social settings. This ‘invisibility paradox’ suggests standard tools may capture relief from acute cancer‐related distress whilst overlooking the enduring psychological challenges that shape long‐term survivorship [49]. Fear of cancer recurrence remained a particularly prevalent and deeply entrenched concern, with survivors describing a constant state of vigilance and ongoing anxiety. This profound anxiety is rooted in the high rate of recurrences, which affects approximately 50% of OC survivors after curative‐intent surgery, with about half of these events occurring in the first postoperative year [50]. Many patients subsequently express a marked preference for frequent imaging‐based surveillance over standard outpatient visits, as they seek greater reassurance and a sense of control over their health [51]. Withdrawal from social activities was also emphasised and appeared multifaceted, often driven by embarrassment, unpredictable gastrointestinal symptoms, and fatigue that hindered participation. These findings collectively underscore how current quantitative assessments may systematically underestimate the true burden of survivorship by equating adaptation with recovery and concealing the profound lifestyle restructuring many survivors are forced to undertake [49, 52, 53]. This limitation is further reinforced by the structure of HRQoL instruments such as the EORTC QLQ‐C30, which allocate considerably fewer items to emotional functioning than to physical and symptom domains, making them less sensitive to the nuanced psychological challenges experienced by survivors [53].

### 4.1 Clinical Implications

Current survivorship care for OC remains misaligned with patient needs [6]. The prevailing surgeon‐led model focuses primarily on oncological surveillance rather than recognising survivorship as an ongoing chronic phase of care that requires comprehensive long‐term management [54]. Qualitative evidence consistently reveals survivors experience care fragmentation and inadequate support once acute treatment concludes [6, 55]. The highly sub‐specialised nature of post‐oesophagectomy management also means that many healthcare professionals outside tertiary centres have limited familiarity with the long‐term care needs, a gap that further contributes to disjointed care and patient distress, as reflected in the qualitative accounts. Addressing this requires targeted education and structural support for primary care providers, particularly in rural settings where general practitioners serve as the key point of continuing care following discharge from tertiary centres [56]. Potential strategies include OC‐specific survivorship care plans with actionable guidance, telementoring networks to build specialist knowledge in community settings [57], formalised shared care protocols [58, 59], and virtual consultation platforms enabling rapid specialist advice for geographically isolated practitioners [60].

The paradigm of survivorship care must also shift towards a structured, multidisciplinary approach that extends well beyond the early post‐operative years [55]. Essential components include ongoing access to specialised dietitians experienced in post‐oesophagectomy nutritional challenges, physiotherapists trained to address deconditioning and fatigue, and psycho‐oncologists equipped to manage associated anxiety and identity disruption. In the OC setting, survivorship care plans should be dynamic and personalised, providing phase‐specific guidance on dietary strategies, symptom management, and clear pathways for seeking help, potentially delivered through accessible digital platforms. Peer support programs are a particularly valuable intervention, as survivors consistently reported that connecting with others who share their experience helps reduce feelings of isolation and fosters a sense of normality [61]. Formalisation of these programs represent a low‐cost, high‐impact strategy that should be actively facilitated through support groups and structured mentoring systems [62]. These strategies align closely with established international survivorship frameworks, including the US Institute of Medicine's survivorship standards [63], the UK's National Cancer Survivorship Initiative [64], and Australia's COSA model [65]. Each emphasises risk‐stratified person‐centred follow‐up, multidisciplinary integration, and proactive management of long‐term treatment effects.

Clinicians should also provide realistic education about the prolonged and often incomplete recovery trajectory following oesophagectomy, emphasising that physical impairment is common and requires proactive management [39]. Rehabilitation through structured, evidence‐based exercise has shown particular promise. The Dutch PERFECT trial and the Irish ReStOre trial have demonstrated that combined aerobic and resistance training can significantly improve cardiorespiratory fitness in long‐term survivors after oesophagectomy, with proven downstream gains in role functioning and global quality of life [66, 67]. Together with the priorities voiced by survivors in the qualitative analysis, these results support integrating structured rehabilitation into survivorship pathways to optimise outcomes.

### 4.2 Limitations

The primary strength of this review is its convergent mixed‐methods design, which integrates quantitative and qualitative evidence to provide a holistic, person‐centered perspective of survivorship. Several limitations must be acknowledged. First, survivorship bias is inherent in the literature as HRQoL data are drawn only from patients alive at follow‐up, systematically excluding those who died from recurrence or postoperative complications and likely underestimating the true population‐level burden of oesophagectomy. Whilst this means the quantitative findings, particularly for long‐term recovery, likely represent a “best‐case scenario”, the implications for both clinical counselling and healthcare resource planning is that the reality for the average patient embarking on this treatment journey is likely more challenging than these aggregated survivor scores suggest. Second, given the lack of pre‐diagnosis data, 'baseline' HRQoL values represent the most recent data obtained prior to surgery rather than pre‐disease states. This means baseline measurements already reflected cancer‐related burden, so recovery trajectories were measured against a compromised starting point rather than genuine health. The current evidence therefore reflects oesophagectomy's impact on HRQoL rather than the complete cancer journey trajectory. Third, whilst this review captures longitudinal data extending to 5 years post‐operatively, we identified a relative paucity of studies with follow‐up at this 5‐year timepoint, highlighting the need for future cohort studies with extended follow‐up to comprehensively characterise long‐term oesophageal cancer survivorship. Fourth, restricting the inclusion of studies to those using EORTC QLQ‐C30 and QLQ‐OES18 instruments, whilst methodologically necessary to enable valid meta‐analysis through standardisation, may have excluded studies that employed other validated HRQoL instruments such as FACT‐G, QOL‐CS, or CaSUN [68, 69, 70]. Fifth, whilst time since surgery was extracted from all qualitative studies, most studies were cross‐sectional with heterogeneous follow‐up periods. This limited the ability to formally stratify the thematic synthesis by discrete postoperative timepoints, though we remained attentive to temporal patterns during coding and contextualised themes within relevant survivorship phases where appropriate. Sixth, the review did not examine potential gender differences in HRQoL trajectories or survivorship experiences. Whilst the included studies demonstrated the expected male predominance reflecting oesophageal cancer epidemiology, most primary studies did not report gender‐stratified outcomes, which may may masque unique survivorship experiences in women. Given emerging evidence that gender and rural location may influence post‐surgical recovery trajectories, future prospective studies should explicitly examine both gender‐specific and geography‐specific survivorship needs with adequate representation from female and rural populations.

## 5 Conclusion

This systematic review reveals that post‐oesophagectomy survivorship constitutes a profound life transformation. Patients must navigate an a ‘new normal’ with chronic physical symptoms and psychosocial distress, set against a triphasic trajectory of HRQoL. Adopting a multidisciplinary model of survivorship care that recognises the need for long‐term management in this cohort could empower individuals to regain a sense of agency in their lives after surgery. Priority areas for development include the proactive management of gastrointestinal symptoms, structured rehabilitation programs, as well as access to psychological services and support networks.

## Author Contributions

Jonathan Sivakumar led the study conceptualisation, methodology, literature search, data curation, and drafting of the original manuscript. Thang Dao, Feras Alnimri, and Qianyu Chen assisted with data extraction, validation, and formal analysis. David S. Liu, Rebekah Laidsaar‐Powell, and Michael W. Hii contributed to interpretation of the findings and critical revision of the manuscript. Cuong Phu Duong oversaw study design, supervision, and final manuscript review. All authors approved the final version.

## Funding

Jonathan Sivakumar is a recipient of and supported by The Alan and Kate Gibson Research Fellowship and the University of Melbourne Melville Hughes Scholarship. Rebekah Laidsaar‐Powell is funded by the Cancer Institute NSW Early Career Fellowship (ID 2022_ECF1457).

## Conflicts of Interest

The authors declare no conflicts of interest.

## Supporting information


Supporting Information S1

## Data Availability

The data that supports the findings of this study are available in the supplementary material of this article.
