# Supplementary material for: Systematic Review and Meta‐Analysis of Patient Experiences of Oesophageal Cancer Survivorship After Oesophagectomy
Source: Psychooncology. 2026 May 11;35:e70482. doi: 10.1002/pon.70482 (PMC13159506; doi:10.1002/pon.70482)
Supplement: Supplementary file 1 — Supporting Information S1 [file PON-35-e70482-s001.docx]

**Electronic supplementary material - Figure S1. Pooled EORTC QLQ-C30 and QLQ-OES18 functional and symptom scores at 12 months following oesophagectomy.**


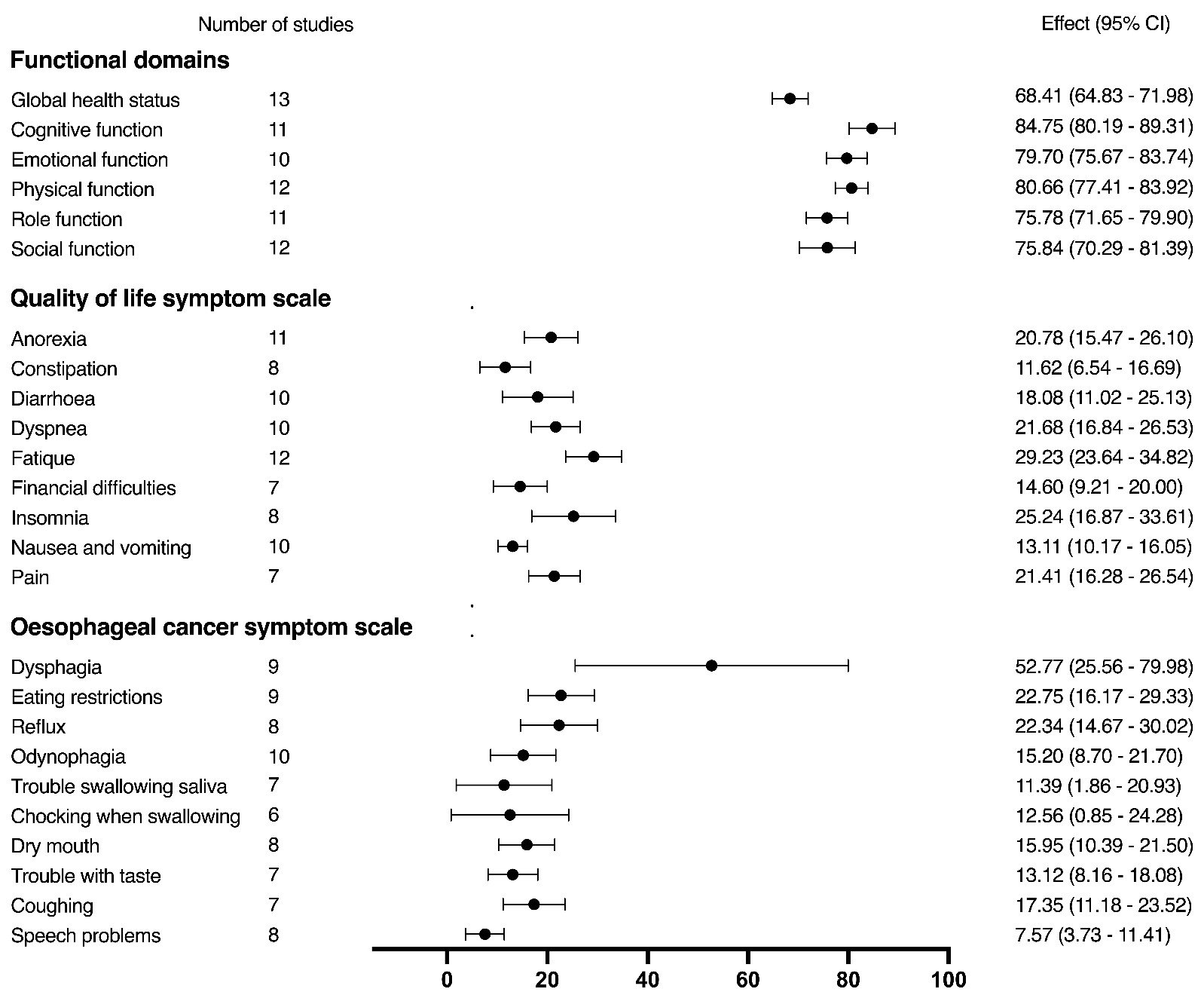


**Electronic supplementary material - Figure S2. Pooled EORTC QLQ-C30 and QLQ-OES18 functional and symptom scores at 5 years following oesophagectomy.**


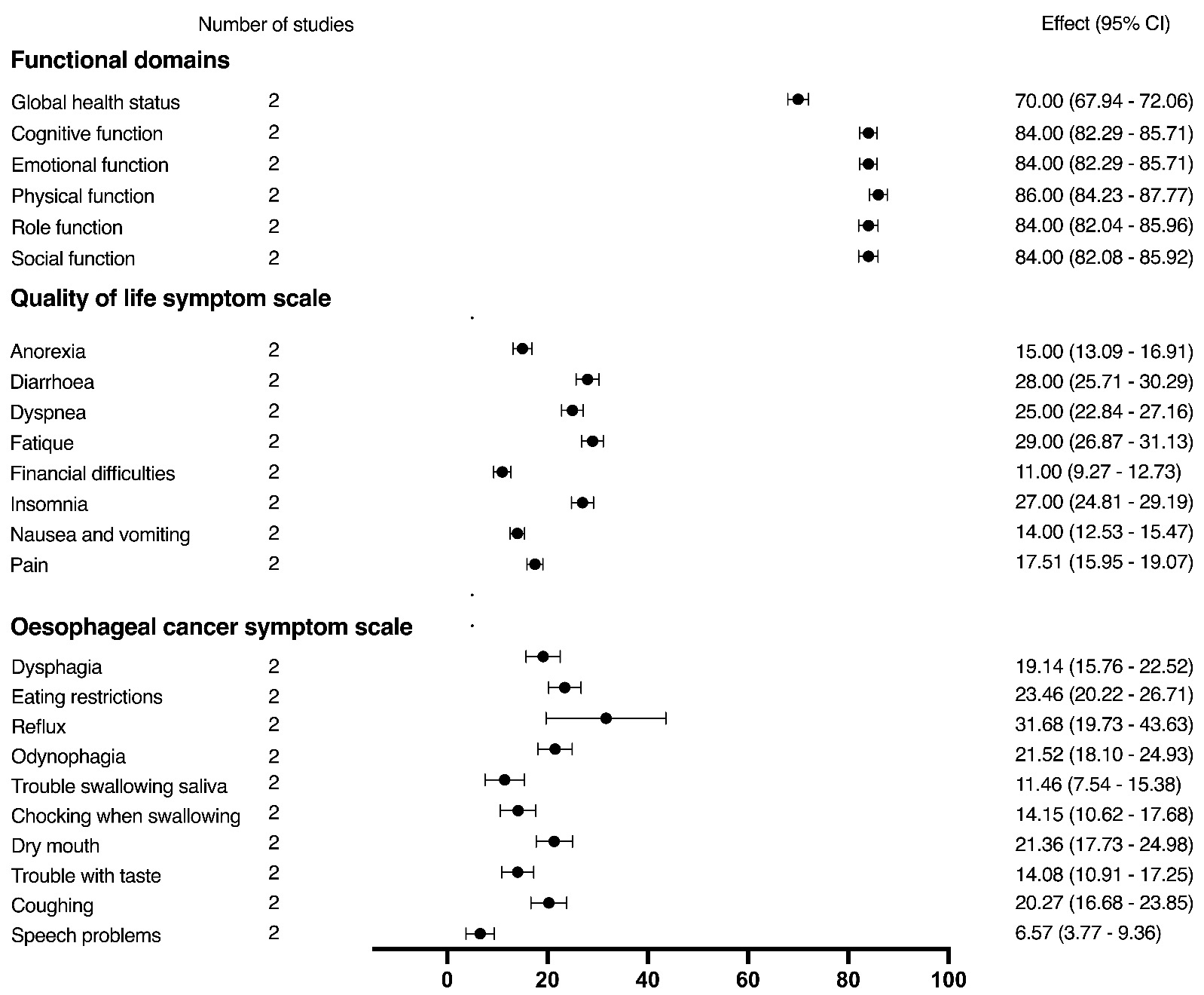


**Electronic supplementary material - Figure S3. Funnel plot assessing small-study effects for 12-month global health status (EORTC QLQ-C30) following oesophagectomy.**


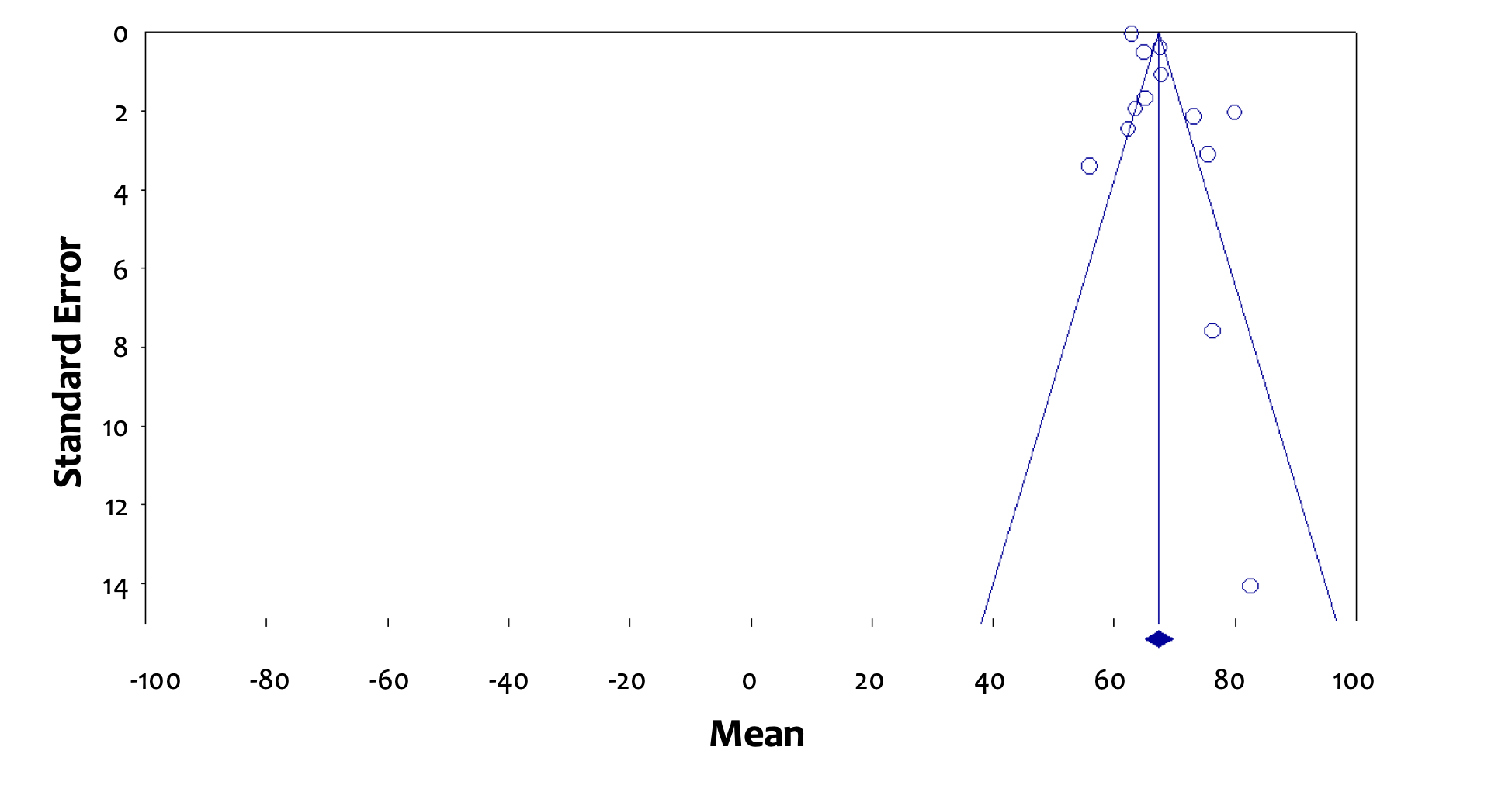


Each point represents an individual study plotting the mean global health status score against its standard error. The vertical line indicates the pooled mean estimate derived from the random-effects model. Visual inspection demonstrates relative symmetry around the pooled estimate, with no clear evidence of substantial small-study effects. Given the single-arm nature of the included studies and the use of pooled mean outcomes rather than comparative effect sizes, formal statistical tests for publication bias were not performed.
